# Factors associated with the research efficiency of clinical specialties in a research-oriented hospital in China

**DOI:** 10.1371/journal.pone.0250577

**Published:** 2021-04-28

**Authors:** Yin Li, Jiachang Li, Baihong Li, Yue Cao, Menghan Liu, Longhao Zhang, Zhi Zeng

**Affiliations:** 1 “Double First-class” Construction Office, West China Hospital, Sichuan University, Chengdu, Sichuan, China; 2 Department of Science and Technology, West China Hospital, Sichuan University, Chengdu, Sichuan, China; 3 Department of Cardiology, West China Hospital, Sichuan University, Chengdu, Sichuan, China; Institute for Advanced Sustainability Studies, GERMANY

## Abstract

Research-oriented hospitals are responsible for medical services tasks, medical education, and scientific research, playing an important role in medical research and application. The research efficiency of a clinical specialty is influenced by factors such as the characteristics of the specialty, the organizational atmosphere, and the clinical director’s leadership. The present study aimed to describe the research efficiency of clinical specialties, explore the factors influencing it, and clarify the argument of co-evolution theory regarding the collaborative development of medical services, education, and research. Logistic regression and multiple linear regression were adopted to estimate the correlation between influencing factors and scientific research efficiency. Hospital H, which is representative of research hospitals in China, was taken as an example. Taking three efficiency values—comprehensive technical efficiency (CTE), pure technical efficiency (PTE), and scale efficiency (SE)—as dependent variables, the independent variables affecting research productivity were statistically analyzed. This study also examined the scientific research efficiency of 41 specialties between 2013 and 2017, and found that the independent variables affected CTE, PTE, and SE to various degrees. Collaborative innovation in medical education and research must be based on clinical research; how to balance medical and teaching quality, and research efficiency requires further discussion. While young people play a major role on the research team because of their creativity and initiatives, which improve CTE and PTE, high-level researchers with better research and leadership abilities lead to the rational allocation and effective utilization of resources, thus improving SE. In 2013–2017, discipline construction focused on scale expansion, resulting in the decline of SE in China. Therefore, this study suggests further improvements for the efficiency of clinical specialties in research hospitals.

## Introduction

Many countries have placed scientific and technological innovations at the core of health and wellness. Increasing investment in medical research has improved health care. The United States has invested more than one-third of total funding in nondefense fields for National Institutes of Health (NIH) research. In the past 10 years, the average annual funding for NIH reached 30 billion dollars and has shown an increasing yearly trend [1]. The United Kingdom plans to invest 15 billion pounds in the research and development of diseases such as cancer within 10 years [2]. Johns Hopkins Hospital (JHH) and Massachusetts General Hospital (MGH) are representative of research-oriented hospitals.

In China, research hospitals began to be built in 2003 [3]; after 17 years, approximately 100 research hospitals were constructed. Given the rapid growth of research hospitals and investments in medical research, how research hospitals’ investigations can be scientifically and rationally evaluated, how research quality and efficiency can be improved, and how the application and transformation of medical research results can be maximized, have become urgent problems in medical and health institutions [4].

Scientific research is a unique creative activity [5], and its influencing factors involve the entire process of scientific research activities [6]. Scientific research efficiency is affected by the external environment and internal factors. In the past, Gu et al. identified the major factors that impact doctoral graduates’ research efficiency (DRP) in China, such as academic status, academic experience, and energy distribution [7]. Edgar et al. identified factors associated with superior research efficiency and demonstrated that autonomy and egalitarianism, along with a strong cultural ethos supporting achievement and individualism, are characteristics of high-functioning departments [8]. Sung et al. identified research manpower and expenses as the critical factors of research hospitals [9]. Hospital specialty, as a subsystem of the hospital system, is also affected by many factors [10]; however, relatively few studies have focused on aspects that influence the research efficiency of hospital specialties.

At present, research on the influencing factors of scientific research efficiency is mainly conducted through questionnaire surveys [11, 12]. In recent years, some studies have begun to use a correlation model to analyze the impact factors on research efficiency. For instance, Wang et al. constructed a multiple regression model to study the factors affecting the scientific research efficiency of 63 universities [13]. Yang applied the decision-making trial and evaluation laboratory (DEMATEL) method to analyze the influence of each factor on the efficiency evaluation of collaborative innovation research in universities. Therefore, it will be more objective to select multiple regression models to explore the influencing factors and more conducive for the improvement of scientific research efficiency.

Different influencing factors affect different objects’ research efficiency; scientific research is a long-term continuous process that is affected by multiple factors. According to Bland, and Center et al., three factors affect scientific research productivity: individual, organizational, and leadership factors [6]. Individual factors include such elements as age, title, status, and researchers’ educational background [14, 15]. Considering that a hospital is a trinity of medical services, education, and research, research activities are affected by medical services and teaching; therefore, organizational factors include medical and teaching quality [16]. The clinical specialty director determines the research direction, resource allocation, and organizational atmosphere of the specialty [17–19]. The leadership factor, therefore, includes clinical director leadership [20].

This study used China’s research-oriented Hospital H as an example. The study first aims to describe the research efficiency of clinical specialties; second, it aims to investigate the characteristics of these factors, and third, it aims to explore the factors influencing research efficiency in hospitals.

## Materials and methods

### Data collection

Hospital H is representative of research hospitals in China and has been ranked first among Chinese hospitals in terms of science and technology influence for seven consecutive years. Hospital H comprises 41 clinical specialties, including nonsurgical specialties (internal medicine diagnosis and treatment specialties), surgical specialties (surgical diagnosis and treatment specialties), and medical technology specialties (testing platform specialties).

The data were obtained from Hospital H’s scientific research data management system, and the extraction time was from 2013 to 2017. We analyzed the value of each specialty’s scientific research input and output, and built a Super-Epsilon-Based Measure (EBM) model based on the decision-making method to evaluate Hospital H’s scientific research efficiency [21]. The Charnes, Cooper, and Rhodes (CCR) radial model and the Banker, Charnes, and Cooper (BCC) non-radial model were used to decompose efficiency into comprehensive technical efficiency (CTE), pure technical efficiency (PTE), and scale efficiency (SE). The values for the 41 clinical specialties were calculated using MaxDea8 software.

### Variable definitions

Based on the three-dimensional factors affecting productivity, and considering the availability and representativeness of the data, we selected eight possible influencing factors: (1) medical quality: comprehensive scores of the annual assessment of specialties’ medical effectiveness (indicators include the rate of four-level operation, the complications rate, the unplanned prehospitalization rate, and the amount of new clinical technology, etc.), medical efficiency (indicators include the degree of increase in per capita medical expenses, the four-level operation ratio, the medical consumables ratio, the average number of hospitalization days, etc.), medical safety and infection (indicators include the number of medical disputes, the adverse events reporting rate, the rate of nosocomial infection outbreaks, etc.), and nursing quality (indicators include workload, post-graduation education, etc.) [22, 23]; (2) teaching quality: comprehensive scores of the annual assessment of specialties’ teaching quality based on teaching achievement, undergraduate education, graduate education, and resident training [24]; (3) discipline construction: the comprehensive reputation ranking of specialties in the annual assessments [25]; (4) specialty operation: the comprehensive scores of the annual assessment of specialty operations and adverse events [26]; (5) proportion of senior professional titles: the ratio of the number of staff with senior professional titles to all staff [27]; (6) proportion of doctors of medicine (MDs) or doctors of philosophy (PhDs): the ratio of the number of MDs or PhDs to all staff [28, 29]; (7) director leadership: a public opinion survey of the clinical director [30, 31]; and (8) age ratio: the ratio of under 40 years to over 40 years, according to the application age requirements of the National Natural Science Foundation of China (NSFC) [32].

### Influencing factor model construction

We used multiple linear regression models to explore the impact of relevant factors on scientific research efficiency. Given that the maximum estimated value of the EBM is 1, it does not meet the condition which states that the value of the dependent variable in the classical linear regression needs to obey the normal distribution. Too many 1s lead to an unstable parameter estimation and a low-test efficiency. In this study, there were 41 decision making units (DMUs) from 2013 to 2017, with a total of 205 observation efficiency values. The observation values of CTE, PTE, and SE that were equal to 1 were 34, 68, and 34, respectively, and the cut-off value was more than 15%. To improve the model’s efficiency, the efficiency value estimated by the Super-EBM model with a window width of 3 was used as the dependent variable. The value of PTE represents the scientific research level and management ability, while the SE value represents resource allocation and utilization [33]. The specialties’ CTE, PTE, and SE were used as dependent variables to clarify the influencing factors’ effects on efficiency. The factors were used as independent variables, and the classical general linear regression was used for parameter estimation.

Owing to the large number of independent variables and a certain degree of multicollinearity, parameter estimation is bound to be unstable. This study adopted a two-way stepwise regression, and the variables were selected according to the Akaike information criterion (AIC) minimum principle. The variables included in the model are shown in Table 5. The statistical software used was R-4.0.2, and the inspection level was 0.05.

### Statistical analysis

IBM1 SPSS1 Statistics software (Armonk, New York, USA) was used to analyze the data, and the participants’ general characteristics were determined by descriptive statistics. Correlations between variables were examined using bivariate correlation. Multiple linear regression analysis was performed to study the variables that can significantly influence specialties’ research efficiency.

## Results

### Descriptive statistics of the Super-EBM results

The mean CTE, PTE, and SE values of all specialties in 2013–2017 were less than 1, and little difference was detected in the annual mean. According to the specialties’ CTE values, CTE > 1 was effective, indicating that the scientific research input had produced a good output—about 19.51%, 12.20%, 17.07%, 14.63%, and 19.51% in 2013, 2014, 2015, 2016, and 2017, respectively (Table 1).

**Table 1:** Descriptive statistics of the specialties’ annual efficiency in 2013–2017.

Options (n = 41)	2013	2014	2015	2016	2017
Mean of CTE	0.74	0.71	0.76	0.72	0.69
Mean of PTE	0.77	0.76	0.92	0.90	0.77
Mean of SE	0.95	0.94	0.88	0.85	0.91
Effective specialties n(%)	8(19.51)	5(12.20)	7(17.07)	6(14.63)	8(19.51)

Table 2 presented a mean CTE of 0.72, with the minimum and maximum being 0.24 and 3.30, respectively. The mean PTE was 0.82, with the minimum and maximum being 0.27 and 3.63, respectively. The mean SE was 0.91, with the minimum and maximum being 0.29 and 1.18, respectively.

**Table 2:** Overall description of the annual average efficiency of the Super-EBM (*n* = 41).

Options	Mean	Min	Max
CTE	0.72	0.24	3.30
PTE	0.82	0.27	3.63
SE	0.91	0.29	1.18

Next, we conducted a hierarchical analysis of the average annual CTE values of the 41 specialties, positioning CTE > 1 as the first level, indicating that the efficiency was effective. Efficiency values in the range of 0.8–1 were positioned as the second level, suggesting that the efficiency was close to effective; those in the range of 0.6–0.8 were positioned as the third level, meaning that the efficiency was lower. Finally, efficiency values below 0.6 were positioned as the fourth level, indicating that the efficiency was particularly low. Table 3 shows that most of the specialties were effective or close to effective. The specialties that were inefficient and far from the effective frontier were in the minority.

**Table 3:** Grade distribution of comprehensive technical efficiency.

CTE classification	Frequency	Percentage
First level	19	46.34%
Second level	6	14.63%
Third level	11	26.83%
Fourth level	5	12.20%

### Descriptive statistics of influencing factors

We collected a total of eight factors that may affect the efficiency of scientific research. We found that the distribution of age ratios in various specialties was relatively balanced, the average proportion of MDs or PhDs in each specialty was 0.45, with the minimum and maximum being 0.03 and 0.90, respectively. Polarization was more obvious. The average proportion of senior professional titles in each department was 0.34. The quantitative scores of the other five indicators are listed in Table 4.

**Table 4:** Characteristics of the influencing factors (*n* = 41).

Variable	M±SD/Frequency (%)	Min	Max
Age Ratio (AR)*			
AR1(0–0.8)	15(36.6%)	-	-
AR2(0.8–1.2)	17(41.5%)	-	-
AR3(>1.2)	9(21.9%)	-	-
Proportion of MDs or PHDs	0.45±0.17	0.03	0.90
Proportion of Senior Professional Titles	0.34±0.1	0.12	0.59
Medical Quality	47.56±2.24	43.05	52.14
Teaching Quality	84.98±3.1	78.14	91.87
Discipline Construction	5.54±0.34	4.50	5.93
Specialist Operations	4.55±0.24	4.04	4.96
Director Leadership	88.19±4.68	75.00	99.09

*AR: Ratio of under 40 years to over 40 years.

#### Relationships between the major variables

The linear correlations between the variables are shown in Table 5. Among them, director leadership showed a significant positive correlation with specialty operations (*r* = 0.369, *p* < 0.05). Teaching quality showed a positive correlation with discipline construction (*r* = 0.419, *p* < 0.01), specialty operations (*r* = 0.444, *p* < 0.01), and director leadership (*r* = 0.497, *p* < 0.01). The proportion of MDs or PhDs and proportion of senior professional titles, and age ratio and proportion of senior professional titles had a statistically obvious linear correlation, and the correlation coefficients were all greater than 0.3 (*p* < 0.05).

**Table 5:** Correlations coefficient matrix between variables.

	medical quality	discipline construction	specialist operations	director leadership	teaching quality	proportion of MDs or PhDs	proportion of senior professional titles	age ratio
medical quality	1	0.068	-0.065	-0.017	-0.065	0.017	0.126	-0.171
discipline construction		1	0.174	0.631	0.419**	0.227	-0.023	-0.205
specialist operations			1	0.369*	0.444**	-0.214	-0.066	-0.224
director leadership				1	0.497**	-0.006	-0.178	-0.206
teaching quality					1	0.199	0.022	-0.004
proportion of MDs or PhDs						1	0.408*	0.167
proportion of senior professional titles							1	0.33*
age ratio								1

*p < 0.05

**p < 0.01.

### Multiple linear regression analysis of influencing factors

In this study, a two-way stepwise regression was conducted to select variables based on the AIC minimum principle. For different dependent variables, the independent variables included in the model are listed in Table 6.

**Table 6:** Variables included in the model.

Dependent variable	Independent variables
CTE	age ratio, teaching quality, medical quality, discipline construction
PTE	age ratio, teaching quality, medical quality, discipline construction
SE	teaching quality, discipline construction, proportion of senior professional titles, proportion of MDs or PhDs, director leadership

We used the least squares method to estimate the parameters of the three models separately; the standardized parameter estimation results are listed in Table 7. When CTE was used as the dependent variable, age ratio 3 significantly improved CTE compared to the other age ratio. Teaching quality had a positive effect on CTE, while medical quality had a negative effect. Under the premise that the influencing factors were statistically significant, the standardized effect size was age ratio 3 > teaching quality > medical quality. When PTE was used as the dependent variable, the effects of age ratio, teaching quality and medical quality were consistent with the CTE model, discipline construction had a positive effect, and the standardized effect size was age ratio 3 > discipline construction = medical quality > teaching quality. In the model with SE as the dependent variable, all influencing factors were statistically significant, with discipline construction exerting a negative effect; the standardized effect size was proportion of senior professional titles > teaching quality = discipline construction > proportion of MDs or PhDs > director leadership.

**Table 7:** Multiple linear regression estimation results.

Independent variables	CTE	PTE	SE
	*R*^*2*^ = 0.27***	*R*^*2*^ = 0.21***	*R*^*2*^ = 0.35***
age ratio ^α^			
age ratio 1 (0–0.8)	-	-	-
age ratio 2 (0.8–1.2)	0.034	0.029	-
age ratio 3 (>1.2)	0.075***	0.074**	-
teaching quality	0.062**	0.056*	0.018*
medical quality	-0.053*	-0.071*	-
discipline construction	0.045	0.071*	-0.018*
proportion of senior professional titles	-	-	0.021**
proportion of MDs or PhDs	-	-	0.017*
director leadership	-	-	0.013*

^α^: Ratio of under 40 years to over 40 years.

*: *P*<0.05;

**: *P*<0.01,

***: *P*<0.001.

-: The independent variable was not included in the model.

In this regression model, the three models’ adjusted *R*^2^ values were 0.27, 0.21, and 0.35, revealing that the 27%, 21%, and 35% variance in efficiency, respectively, could be explained by the seven aforementioned variables. Compared with the zero model, the *p* values were all less than 0.001. The three models’ residual graphs had no obvious change rules. Except for individual extreme values, they were close to white noise, indicating that the establishment of this model and the results are reasonable.

## Discussion

### Efficiency and specialties

The present study found that the mean CTE values (the proportion of research effective specialties) were 0.74 (19.51%), 0.71 (12.20%), 0.76 (17.07%), 0.72 (14.63%), and 0.69 (19.51%) for 2013, 2014, 2015, 2016, and 2017, respectively. A five-year comparison could imply that each year, the proportion of research effective specialties was in a stable fluctuation of around 15%. In total, 19 specialties were effective, and the proportion of research effective specialties was 46.34% in 2013–2017. Scientific research is a continuous, long-term process. The annual proportion of research effective specialties was relatively small, but after five years of accumulation, the output of research results increased significantly, and the overall efficiency saw a substantial increase. The proportion of research effective specialties increased to 46.34%. Regarding research specialty efficiency at Peking University People’s Hospital, Ling found that effectiveness had reached 38.90% in 2010–2015 [34]. Hospital H is a research-oriented hospital that was ranked first in scientific research among Chinese hospitals, of which 15 specialties were in the top five, and 24 specialties were in the top ten. Both, Hospital H and Peking University People’s Hospital are famous research hospitals in China, and demonstrate that approximately 40% of specialties account for the overall effectiveness for five years of input. According to Guironnet, in terms of the research efficiency of 165 American universities with a hierarchical data envelopment analysis (DEA) model, 38 units were efficient in 2012, equivalent to 23% [35], which exceeded that of medical schools in China. This shows that developed countries have more advantages in terms of short-term input and output. Therefore, specialties’ research efficiency should be brought to hospital managers’ attention, especially with regard to short-term efficiency. Further studies should explore strategies and measures to improve research efficiency.

### Collaborative innovation

The results of the multiple linear regression model demonstrated that research efficiency and teaching quality are positively correlated. For instance, graduate students, as the main driving force on a research team, received training from and conducted research with the team, and their research productivity was apparently influenced by teaching quality.

The synergy theory on medical service and research collaboration assumes that research results will improve the quality of medical services [36]. However, due to the competition between clinical service and scientific research for resources, including people and time, it is difficult for clinicians to balance clinical work and medical research, as they are expected to be effective at curing patients while focusing on research. In 2013–2017, domestic medical research in China attached importance to basic research, and clinical research was relatively weak. In addition, the clinical application of basic research results generally took a long time. Although there is considerable research output based on basic research, it cannot directly lead to an outstanding quality of medical services. In conclusion, medical quality had a negative effect on medical research efficiency in China during 2013–2017. Our study suggests that medical research should focus on solving clinical problems; therefore, the collaborative innovation of medical services, medical education, and medical research must be based on clinical research. Questions on how to balance medical and teaching quality, and research efficiency require further discussion.

### Efficiency and human resources

Taking the 40 years as the limit [37], an age ratio of greater than 1.2 can significantly improve the efficiency of scientific research. This shows that young people’s role in research teams is more important because their creativity drives technological innovation, thus resulting in improved PTE and CTE. The proportion of senior professional titles has the greatest positive effect on SE, and the allocation and utilization of resources is mainly based on researchers with senior professional titles; therefore, the higher the proportion, the more the available resources. The proportion of staff with doctoral degrees or senior professional titles is a symbol of academic qualifications [38, 39], and a high proportion indicates a high-level research ability; in addition, the use of resources is more effective, positively affecting SE. Similarly, the clinical director’s leadership is reflected in their ability to organize and mobilize [40, 41]. Reasonable, effective resource allocation will help improve SE. Discipline construction has a positive effect on PTE, as it promotes the introduction of advanced technology and innovation in management mechanisms. The better the discipline construction, the higher the PTE. However, discipline construction has a negative effect on SE. During 2013–2017 in China, in order to enhance the academic reputation, discipline construction of the specialties focused on scale construction, which resulted in an improved academic reputation; however, it ignored efficient resource utilization.

### Limitations

A limitation of our study is the incompleteness of the factors. Research is a long-term, complex, productive activity and is affected by many factors. Based on the theory of scientific research productivity, we screened eight indicators that might affect specialties’ research efficiency in hospitals and also considered the feasibility of indicator data acquisition.

According to the models’ adjusted *R*^2^ (0.27, 0.21, 0.35), the selected variables in the three models had a certain degree of explanatory strength for the changes in efficiency; the strength was relatively small, however, suggesting that the specialties’ efficiency values maybe were also determined by other factors such as physician–nurse ratio, number of beds, medical research time ratio, teaching, and research motivation. We will continue to explore other influencing factors and improve the influencing factor system to better reveal the influencing mechanism and effectiveness.

## Conclusion

The five-year effectiveness ratio of research hospitals’ specialties is approximately 50% in China. In terms of research efficiency, more than half of the specialties were ineffective, indicating input redundancy. The annual scientific research efficiency in developed countries is significantly higher than that of China, owing to the advantages of technology and other mechanisms. Regarding the abovementioned factors, the results demonstrated the co-evolution of teaching and research. Collaborative innovation in medical quality and research must be based on clinical research. Methods for balancing medical and teaching quality, and research efficiency require further discussion. While young people are creative with strong initiatives, high-level researchers have better scientific research abilities; better leadership abilities lead to rational allocation and effective utilization of resources, which would result in improvements in efficiency. Discipline construction should not focus on the construction of scale in the next few years. The study’s findings provide ideas for further improving the research efficiency of clinical specialties in research-oriented hospitals.

## Supporting information

S1 Data (XLSX)

## References

[1] Pawlyk A. NIH funding opportunities and resources. Protein Sci. 2017;26:205-.

[2] Brumfiel G. UK research centre born amid cuts. Nature. 2010;465(7301):996-. 10.1038/465996b 20577181

[3] CB J, ZW X, BH Y, YP X, YH L, YJ Z. Invigorate the hospital with science and education and establish research-oriented hospital. Chinese Journal of Medical Research Management. 2003;01:3.

[4] Yao J, Gao T. Theoretical Interpretation of Chinese Research Hospitals (5) Scientific Research in Research Hospitals. Journal of China Research Hospital. 2015;2(06):53–63.

[5] Hardre P, Cox M. Evaluating faculty work: expectations and standards of faculty performance in research universities. Res Pap Educ. 2009;24(4):383–419.

[6] Bland CJ, Center BA, Finstad DA, Risbey KR, Staples JG. A theoretical, practical, predictive model of faculty and department research productivity. Acad Med. 2005;80(3):225–37. 10.1097/00001888-200503000-00006 15734804

[7] Gu JB, Lin Y, Vogel D, Tian W. What are the major impact factors on research performance of young doctorate holders in science in China: a USTC survey. High Educ. 2011;62(4):483–502.

[8] Edgar F, Geare A. Factors influencing university research performance. Stud High Educ. 2013;38(5):774–92.

[9] Sung KK, Lim DO. A Study of the Critical Factors on Research Capability of Research-driven Hospital. Journal of Health Informatics and Statistics. 2016;41(4):428–35.

[10] Caminiti C, Iezzi E, Ghetti C, De’Angelis G, Ferrari C. A method for measuring individual research productivity in hospitals: development and feasibility. Bmc Health Serv Res. 2015;15. 10.1186/s12913-015-1130-7 26467208 PMC4607003

[11] Kong W, Huang QM. Analysis of influence factors of scientific research performance in medical school. Journal of Shanghai Jiaotong University (Medical Edition). 2008;28(9):1174–7.

[12] EX H, Rong Q, Yang Y, Jing S. An Empirical Study on the Influencing Factors of Scientific Research Team’s Innovation Performance from the Perspective of Collaborative Innovation. Human Resources Development in China. 2016;15:15–27.

[13] wx w, yu w. Research on Scientific Research Performance and Influencing Factors of Universities——Taking Universities Directly Under the Ministry of Education as an Example. Friends of Accounting. 2017(10):109–14.

[14] Gerrits RG, Mulyanto J, Wammes JD, van den Berg MJ, Klazinga NS, Kringos DS. Individual, institutional, and scientific environment factors associated with questionable research practices in the reporting of messages and conclusions in scientific health services research publications. Bmc Health Serv Res. 2020;20(1). 10.1186/s12913-020-05624-5 PMC7469341 32883306

[15] Zhao WF, Liu WJ, Zhang RY. New methods to quantify an individual’s scientific research output based on h-index and various factors. Qual Quant Methods L. 2016:221–34.

[16] Schneider AM, Oppel EM, Winter V. Explaining variations in hospitals’ use of strategic human resource management: How environmental and organizational factors matter. Health Care Manage R. 2021;46(1):2–11. 10.1097/HMR.0000000000000242 30908315

[17] Cassar V, Bezzina F, Buttigieg SC. The relationship between transformational leadership and work attitudes Comparing mediating influences of social identity and the psychological contract. Leadership Org Dev J. 2017;38(5):646–61.

[18] Pritt BS, Bowler CA, Theel ES. Fellowship Training for the Future Clinical Microbiology Laboratory Director. Clin Lab Med. 2020;40(4):521–33. 10.1016/j.cll.2020.08.009 33121620

[19] Strauss JS, Livingood CS. Weary, Peyton E., Elected to Leadership Position in the American-Board-of-Medical-Specialties. J Am Acad Dermatol. 1988;18(6):1354–5. 3290289 10.1016/s0190-9622(88)80111-7

[20] Salvador J. Leadership and Innovation in the Navigation Plan of the Endocrinology and Nutrition Specialty. Endocrinol Diab Nutr. 2019;66(5):275–7. 10.1016/j.endinu.2019.03.007 31006516

[21] Sun YY, Hou GL, Huang ZF, Zhong Y. Spatial-Temporal Differences and Influencing Factors of Tourism Eco-Efficiency in China’s Three Major Urban Agglomerations Based on the Super-EBM Model. Sustainability-Basel. 2020;12(10).

[22] Wu SW, Chen T, Pan Q, Wei LY, Xuan Y, Li C, et al. Establishment of a Comprehensive Evaluation System on Medical Quality Based on Cross-examination of Departments within a Hospital. Chinese Med J-Peking. 2017;130(23):2872–7. 10.4103/0366-6999.219163 PMC5717868 29176146

[23] Horbar JD. The Vermont-Oxford Neonatal Network—Integrating Research and Clinical-Practice to Improve the Quality of Medical-Care. Semin Perinatol. 1995;19(2):124–31. 10.1016/s0146-0005(05)80032-1 7604303

[24] Peiris-John R, Selak V, Robb G, Kool B, Wells S, Sadler L, et al. The State of Quality Improvement Teaching in Medical Schools: A Systematic Review. J Surg Educ. 2020;77(4):889–904. 10.1016/j.jsurg.2020.01.003 32057742

[25] Huang ZK, Liang YF. Research of data mining and web technology in university discipline construction decision support system based on MVC model. Libr Hi Tech. 2020;38(3):610–24.

[26] Abe TK, Beamon BM, Storch RL. Operations research applications in hospital operations: Part III. IIE Transactions on Healthcare Systems Engineering. 2016;6(3):179–91.

[27] Yang S. Misunderstandings to Double Professional Titled Teachers of University & Their Ability and Quality Qualifications. International Conference on Education and Development (Iced 2016). 2016:168–70.

[28] Skinnider MA, Twa DDW, Squair JW, Rosenblum ND, Lukac CD, Grp CMPPI. Predictors of sustained research involvement among MD/PhD programme graduates. Med Educ. 2018;52(5):536–45. 10.1111/medu.13513 29532953

[29] Afzal S, Qaiser F, Ijaz SH, Sarfraz H, Saeed I, Sheikh S, et al. To Assess Evaluation System in Public Medical Institutions at Under-Graduate Level—The Students’ Perspective. Pak J Med Health Sci. 2016;10(4):1214–8.

[30] Roy KM, Roberts MC, Stewart PK. Research productivity and academic lineage in clinical psychology: Who is training the faculty to do research? J Clin Psychol. 2006;62(7):893–905. 10.1002/jclp.20271 16688686

[31] Skinner EH, Williams CM, Haines TP. Embedding research culture and productivity in hospital physiotherapy departments: challenges and opportunities. Aust Health Rev. 2015;39(3):312–4. 10.1071/AH14212 25774754

[32] Bird S, Michelin MJ, Toseland PA, Townsend J, Tutt PW. Impact of Staffing Structure on Hospital Laboratory Productivity and Cost. Lancet. 1992;339(8798):934–5. 10.1016/0140-6736(92)90976-a 1348329

[33] Li JK, Zhang J, Gong LT, Miao P. Research on the Total Factor Productivity and Decomposition of Chinese Coastal Marine Economy: Based on DEA-Malmquist Index. J Coastal Res. 2015:283–9.

[34] Tao LGWDWBLYZHX. Performance Analysis and Development Countermeasures of Clinical Research Based on DEA. Chinese Journal of Medical Research Management. 2017;30(03): 177–80.

[35] Guironnet JP, Peypoch N. The geographical efficiency of education and research: The ranking of US universities. Socio-Econ Plan Sci. 2018;62:44–55.

[36] De Vito EL. The scientific production of public hospitals. Medical care, teaching and research: a utopian virtuous circle? Medicina-Buenos Aire. 2018;78(3):220–1. 29940555

[37] Habib M, Abbas J, Noman R. Are human capital, intellectual property rights, and research and development expenditures really important for total factor productivity? An empirical analysis. Int J Soc Econ. 2019;46(6):756–74.

[38] Kumar PD. Research Productivity of MD-PhDs. Am J Med. 2009;122(7):E9–E. 10.1016/j.amjmed.2009.01.024 19559155

[39] Kwan JM, Daye D, Schmidt ML, Conlon CM, Kim H, Gaonkar B, et al. Exploring intentions of physician-scientist trainees: factors influencing MD and MD/PhD interest in research careers. Bmc Med Educ. 2017;17. 10.1186/s12909-017-0954-8 28697782 PMC5505137

[40] Allison S, Goodall AH, Bastiampillai T. Research leadership: should clinical directors be distinguished researchers? Australas Psychiatry. 2016;24(3):249–51. 10.1177/1039856215612988 26498150

[41] Searle A, Scott JK, Sandy J, Ness A, Waylen A. Clinical directors’ views of centralisation and commissioning of cleft services in the UK. Bmc Oral Health. 2015;15. 10.1186/1472-6831-15-12 PMC4320534 25608950

